# Korean Traditional Medicine (*Jakyakgamcho-tang*) Ameliorates Colitis by Regulating Gut Microbiota

**DOI:** 10.3390/metabo9100226

**Published:** 2019-10-14

**Authors:** Seung-Ho Seo, Tatsuya Unno, Seong-Eun Park, Eun-Ju Kim, Yu-Mi Lee, Chang-Su Na, Hong-Seok Son

**Affiliations:** 1 School of Korean Medicine, Dongshin University, Naju 58245, Korea; Seosa@dsu.kr (S.-H.S.); seong9525@dsu.kr (S.-E.P.); yci3431@dsu.kr (E.-J.K.); lym@dsu.kr (Y.-M.L.); 2 School of Life Sciences, Faculty of Biotechnology, SARI Jeju National University, Jeju 63243, Korea; tatsu1207@gmail.com; 3 Subtropical/tropical Organism Gene Bank Jeju National University, Jeju 63243, Korea

**Keywords:** *Jakyakgamcho-tang*, inflammatory bowel disease, microbiota, metabolomics

## Abstract

The objective of this study was to examine the anti-colitis activity of *Jakyakgamcho-tang* (JGT) in dextran sulfate sodium (DSS)-induced colitis and explore changes of the gut microbial community using 16S rRNA amplicon sequencing and metabolomics approaches. It was found that treatment with JGT or 5-aminosalicylic acid (5-ASA) alleviated the severity of colitis symptoms by suppressing inflammatory cytokine levels of IL-6, IL-12, and IFN-γ. The non-metric multidimensional scaling analysis of gut microbiome revealed that JGT groups were clearly separated from the DSS group, suggesting that JGT administration altered gut microbiota. The operational taxonomic units (OTUs) that were decreased by DSS but increased by JGT include *Akkermansia* and *Allobaculum*. On the other hand, OTUs that were increased by DSS but decreased by 5-ASA or JGT treatments include Bacteroidales S24-7, Ruminococcaceae, and Rikenellaceae, and the genera *Bacteroides*, *Parabacteroides*, *Oscillospira*, and *Coprobacillus*. After JGT administration, the metabolites, including most amino acids and lactic acid that were altered by colitis induction, became similar to those of the control group. This study demonstrates that JGT might have potential to effectively treat colitis by restoring dysbiosis of gut microbiota and host metabolites.

## 1. Introduction

Inflammatory bowel disease (IBD) is a chronic disease that has two main types: Crohn’s disease and ulcerative colitis. The cause of IBD is unclear. There is also no definitive treatment for IBD. Currently, most IBD treatments require lifelong drug treatment such as steroids, antibiotics, thiopurines, aminosalicylates, and anti-tumor necrosis factor (TNF)-alpha antibodies [1,2,3,4,5]. Among them, 5-aminosalicylic acid (5-ASA) is known as a highly effective treatment to treat IBD, including Crohn’s disease and ulcerative colitis [6]. However, the use of these drugs for a long period is associated with a variety of side effects, including diarrhea, nausea, vomiting, headache, and osteoporosis [7]. In severe cases, patients may experience recurrence, drug resistance, and new IBD symptoms [8,9]. Therefore, new approaches are needed to treat IBD safely and effectively.

*Jakyakgamcho-Tang* (JGT) is a Korean traditional herbal medicine that is composed of *Glycyrrhiza uralensis* and *Paeonia lactiflora*. JGT has been traditionally used as herbal medicine in Korea for gastrointestinal inflammation showing symptoms of abdominal pain, abdominal cramps, and so forth. The major active constituents in JGT are gallic acid, oxypaeoniflorin, albiflorin, paeoniflorin, liquiritin, benzoic acid, isoliquiritin, ononin, benzoylpaeoniflorin, and glycyrrhizin [10]. Glycyrol isolated from *Glycyrrhiza uralensis* exerts anti-inflammatory effects [11]. The total glucosides of *Paeonia lactiflora* also exhibit immune regulatory and anti-inflammatory effects [12,13]. Our previous study has shown that acute colitis symptoms could be alleviated by JGT administration [10]. Therefore, JGT may be a promising therapeutic candidate for colitis.

The human gut contains various microbial organisms known as the microbiota with high density and complexity [14,15,16]. The disruption of the symbiotic state between the host and gut microbiota is a significant factor in the etiology of IBD [17]. Numerous studies reported that gut microbiota are involved in the development and treatment of IBD [18,19,20]. The compounds that are ingested as food or drugs can affect the microbial community in the gut. For example, Maier et al. [21] recently reported the change of human gut bacteria by non-antibiotic drugs.

JGT consists of complex mixtures of many compounds, including fiber, polyphenol, and polysaccharide. These compounds are well known to bring beneficial effects to the host by affecting metabolic activities of its gut microbiota. Many previous studies have shown that natural products exert pharmacological effects by modulating the composition of gut microbiota [22,23,24]. Likewise, the potential beneficial effects of JGT on colitis may be partially related to its interaction with gut microbiota. Therefore, the effect of herbal extract on colitis should be examined by focusing on the interaction between the herbal extract and gut microbial community. However, the anti-inflammatory effects of JGT on colitis via changes of the gut microbial community have not been reported yet. Thus, the objective of this study was to examine the anti-colitis activity of a JGT extract in dextran sulfate sodium (DSS)-induced colitis and explore changes of the gut microbial community using 16S rRNA amplicon sequencing. GC/MS-based metabolomics were also employed in a profiling mode to reveal changes in key metabolites of the gut and serum.

## 2. Materials and Methods

### 2.1. Subsection

Male Sprague Dawley rats (Samtako Bio Korea, Osan, Korea) weighing approximately 160–170 g were raised in a constant environment (12 h light/dark cycles, 24 ± 1 °C temperature, 60 ± 5% humidity). The animals were provided free access to standard rat chow and water in their cages. There were five experimental groups according to treatments: (1) without any action, the control group (*n* = 9); (2) DSS (Sigma Aldrich, St. Louis, MO, USA)-induced colitis, the DSS group (*n* = 12); (3) 100 mg/kg 5-aminosalicylic acid (5-ASA; Sigma Aldrich, St. Louis, MO, USA) treatment after colitis induction, the 5-ASA group (*n* = 9); (4) 100 mg/kg JGT treatment after colitis induction, the JGT-A group (*n* = 9); (5) 150 mg/kg JGT treatment after colitis induction, the JGT-B group (*n* = 9). These doses of JGT (100–150 mg/kg) were chosen based on existing clinical doses (Hanpoong Pharmaceutical Company, Seoul, Korea). The rat experiment was conducted in accordance with guidelines of the Ethics Committee of Dongshin University after obtaining approval for this study (approval number: 2018-01-04). The major components of the JGT extract have been presented in a previous paper [10].

### 2.2. Colitis Induction and Treatment

The rats used for the experiment had an adaptation period of three days. Colitis was induced by giving rats 3% DSS (dissolved in drinking water) for 7 days. Thereafter, they were treated with 5-ASA and JGT for 3 days. This process was carried out in four cycles. These rats were checked daily for colitis development by monitoring their body weight, gross rectal bleeding, stool consistency, and survival. The rats fasted for 2 h before sacrifice. Serum, stool, and colon tissues were collected for analysis. The detailed experimental design is illustrated in Supplementary Figure S1.

### 2.3. Histopathological Analysis and Cytokine Quantification

The distal part of the gut was embedded in paraffin, sliced to a thickness of 6 μm, and stained with hemotoxylin and eosin (HE). The inflammation and tissue damage were observed in a blinded manner using a light microscope (80i, Nikon, Tokyo, Japan). The levels of cytokines (TNF-α, IL-6, IL-10, IL-12, and IFN-γ) in the gut tissues were measured using a respective enzyme-linked immunosorbent assay (ELISA) kit (Invitrogen, Carlsbad, CA, USA) as inflammation indicators. The ELISA plates were read at a wavelength of 450 nm using a Spectramax plate reader (M2, Molecular Devices, San Jose, CA, USA).

### 2.4. Fecal Microbiome Analysis

The feces were collected on the last day of the experiment. The total DNA was extracted using a QIAamp^®^ PowerFecal^®^ DNA kit (QIAGEN, Hilden, Germany). The V4 hypervariable region of the 16S rRNA gene was amplified for the microbial community analysis. The library for Illumina MiSeq (250 bp × 2) was constructed by a two-step PCR and sequenced at Macrogen Inc. (Seoul, Korea) according to the manufacturer’s instructions.

The sequence data was processed using Mothur software [25] according to the MiSeq SOP (https://www.mothur.org/wiki/MiSeq_SOP). In brief, the paired-end reads were assembled with make contigs and aligned to the SILVA database (release 128) [26], the singleton reads were removed, and the errors were corrected with pre.cluster. The chimeric sequences were removed using VSEARCH [27] and a taxonomic classification was done based on the greengenes database (version 13_8_99) [28]. Clustering was performed using opti.clust algorithm [29] to assign operational taxonomic units (OTUs). The taxonomic classification of each OTU was done using classify.otu Mothur subroutine. The distribution of OTUs were analyzed using nonmetric multidimensional scaling (NMDS) and the analysis of molecular variance (AMOVA) was applied to detect significant clustering of different treatment groups in NMDS. The linear discriminant analysis effect size (LEfSe) method was used as a Galaxy module (http://huttenhower.sph.harvard.edu/galaxy/) to determine the features most likely to explain the differences between the different groups (LDA > 3, *p* < 0.05) [30]. The statistical differences between the pairwise comparisons were calculated using the Mann-Whitney *U* test. Statistical significance was defined at *p* < 0.05.

### 2.5. Metabolites Analysis

The sample preparation protocol for the GC/MS analysis was the same as described previously [31,32]. Briefly, 300 μL of methanol [methanol:water (7:3)] was added to 100 μL of serum sample or a freeze dried fecal sample (100 mg) and then vigorously extracted. After centrifugation, the supernatant was dried, methoxymated, and trimethylsilylated. In order to minimize systematic variations, all samples were detected in a random order. For detection, the derived sample (600 μL) was injected into a Rtx-5MS fused silica capillary column (30 m × 0.25 mm ID, J&W Scientific, CA) using a split model (1:10) in a Shimadzu QP2020 GC/MS system (Kyoto, Japan). The initial temperature of the GC oven was programmed at 60 °C for 1 min. It was then increased to 300 °C at a rate of 10 °C/min and held for 10 min. The MS was operated in full-scan mode at a range of *m*/*z* 50–600. The injector, ion source, and transfer-line temperatures were maintained at 250, 230, and 280 °C, respectively. The ionization energy in electron impact ionization (EI) mode was set at 70 eV.

The GC/MS data were pre-processed using XCMS software (https://xcmsonline.scripps.edu) for noise reduction, baseline correction, and alignment. The peak intensities of the obtained features were normalized against an internal standard (methyl stearate) before performing multivariate analyses. For the multivariate analyses, the GC/MS data files were imported into SIMCA-P 15.0 software (Umetrics, Umea, Sweden). The statistical analysis was determined using GraphPad Prism 6 (GraphPad Software, Inc., San Diego, CA, USA). The identification of metabolites was performed by comparing their mass spectra with NIST 14.0.

### 2.6. Correlation Analysis

The associations among the metabolites, gut microbes, and cytokines were assessed by Spearman’s rank correlation analysis. A *p* value of less than 0.05 was considered statistically significant. The false discovery rate at 5% was applied to all tests to correct for multiple testing.

## 3. Results and Discussion

### 3.1. JGT Weakens Symptoms of DSS-Induced Colitis

**Figure 1:**
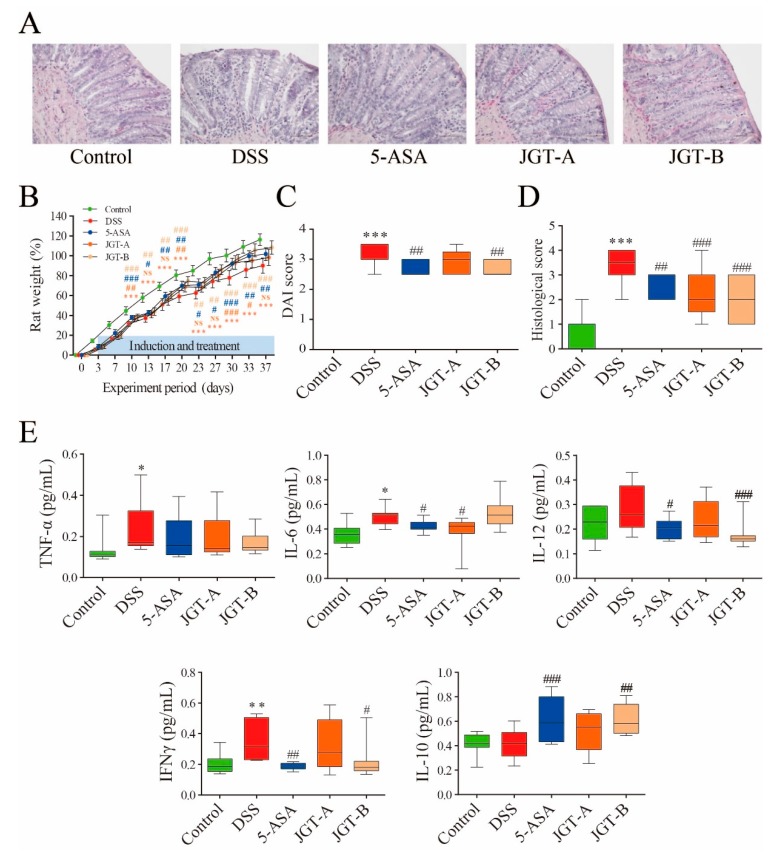
The effect of *Jakyakgamcho-tang* (JGT) on inflammation of gut tissue in colitis rat. (**A**) Representative images by hemotoxylin and eosin (HE) staining displaying colon segments on the day of sacrifice. (**B**) Weight, (**C**) disease activity index (DAI) value, and (**D**) histological score. (**E**) Levels of TNF-α, IL-6, IL-10, IL-12, and IFN-γ. The statistical outcome was summarized within the figure (*t* test: *p* value). Symbols (*) indicate significant difference between control and dextran sulfate sodium-induced colitis (DSS) group (*, *p* < 0.05; **, *p* < 0.01; ***, *p* < 0.001). Symbols (#) indicate significant difference between DSS group and treatment group (5-ASA or JGT) (#, *p* < 0.05; ##, *p* < 0.01; ###, *p* < 0.001).

To assess effects of JGT on colitis induced by DSS, clinical symptoms were determined (Figure 1). In this study, the pattern of symptoms observed after colitis induction was in agreement with those found in previous studies using this animal model [33]. The HE-stained colorectal sections showed that the rats from the DSS group exhibited damage to the mucosa and submucosa with inflammation cell infiltration (Figure 1A). A significant decrease in body weight was observed in the DSS group. The rats in the 5-ASA group and JGT groups experienced less weight loss compared to those in the DSS group (Figure 1B). The disease activity index (DAI) score (calculated by using body weight loss, stool consistency, and blood in stool) increased significantly after the DSS intake, whereas it was markedly attenuated in the 5-ASA group and JGT groups at 37 days after colitis induction (Figure 1C). In addition, the 5-ASA group and JGT groups exhibited obvious recovery of normal colon tissue structures (Figure 1A,D).

DSS-induced colitis begins with the disruption of the gut epithelial barrier function that leads to hypersensitive innate immune cells to gut microbiota [34]. This event provokes the release of pro-inflammatory cytokines at the mucosal level. IL-6, IL-10, IL-12, TNF-α, and IFN-γ play a pivotal role in the induction and recovery of colitis [35,36]. The effects of JGT on the production of cytokines are presented in Figure 1E. The levels of TNF-α, IL-6, and IFN-γ significantly increased in the gut tissues of the DSS group compared to those in the control group. The administration of 5-ASA and JGT suppressed the accumulation of IL-6, IL-12, and IFN-γ in the colon tissues of rats induced by DSS. The level of IL-10 was significantly up-regulated in the 5-ASA treated rats, consistent with the results of Zhou et al. [37]. Although essential targets and mechanisms of IL-10 action are incompletely understood, the anti-inflammatory signal of IL-10 is involved in maintaining intestinal homeostasis [38]. In our results, the decreased levels of pro-inflammatory cytokines in JGT-treated groups indicated that JGT could effectively ameliorate DSS-triggered colonic inflammation. A previous study has shown that paeoniflorin, the principle component of *Paeonia laciflora*, can inhibit LPS-stimulated TNF-α and IL-1β release and promote LPS-induced IL-10 production [39]. Yuan et al. [40] found that glycyrrhizin, the main component of *Glycyrrhiza glabra*, can reduce colonic injury with the suppression of NF-κB, TNF-α, and intercellular adhesion molecule-1 in affected mucosa. Similarly, Kudo et al. [41] reported that glycyrrhizin treatment can significantly reduce the expression levels of pro-inflammatory cytokines and chemokines including IL-1β, IL-6, TNF-α, cytokine-induced neutrophil chemoattractant-2, and monocyte chemoattractant protein-1 in inflamed mucosa.

### 3.2. JGT Treatment Changes Community Structure of Gut Microbiota

The gut microbial community plays an important role in the onset and recovery of colitis. Dysbiosis of the gut bacterial community is involved in patients with colitis and animal models of experimental colitis [42]. To analyze the structural changes in the gut microbiota of the five experimental groups, 16S rRNA gene amplicon sequencing was performed. A total of 1,359,277 Miseq reads from five groups were obtained to investigate the bacterial community differences across the groups. The alpha diversity results are shown in Figure S2. A comparison of ecological indices indicated that the DSS group significantly decreased species evenness (*p* < 0.01), whereas no significant differences in species richness was observed. The 5-ASA or JGT did not have a significant effect on species richness or evenness. 

**Figure 2:**
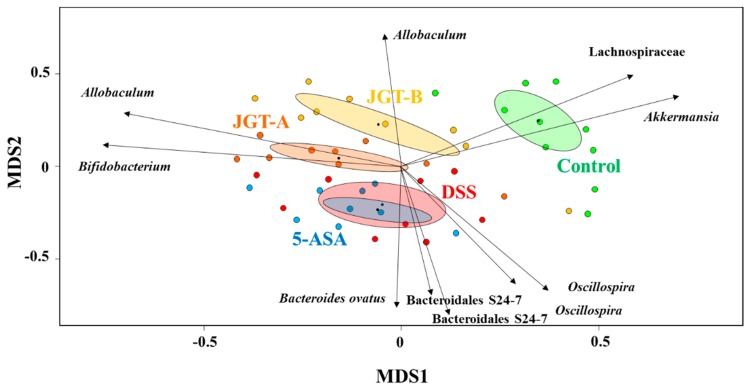
Biplot from a non-metric multidimensional scaling (NMDS) (stress value = 0.299, *R*^2^ = 0.64) with 48 sample sites and species vectors. Ten most correlated operational taxonomic units (OTUs) were indicated with arrows representing the strength and direction of the correlation.

To explore the dissimilarities between the samples, AMOVA was conducted based on the Bray-Curtis distance, and these distances were ordinated and visualized via an NMDS biplot (Figure 2). The DSS group and 5-ASA group did not differ significantly, while all groups appeared to be significantly different from each other (*p* < 0.05). These results suggest that 5-ASA administration does not shift gut microbiota despite the beneficial effects of 5-ASA, while JGT administration shifted gut microbiota. The results in Figure 2 indicate that these shifts are associated with physiologically important bacteria. For example, the treatment of DSS reduced the abundance of *Akkermansia* that resides in the intestinal mucosal layer, likely due to the tissue damages caused by DSS. On the other hand, the abundance of probiotics, *Bifidobacterium*, and the short chain fatty acid producer *Allobaculum* were significantly associated with the JGT treatment. In addition, the higher concentration of the JGT-B group was associated with the increase of *Adlercreutzia*, some species of which were previously reported to utilize isoflavone to produce equol in the human gut [43]. In contrast, the JGT treatment negatively associated with the genus *Oscillospira* and the family S24-7.

### 3.3. JGT Treatment Restores the Changed Microbiota Composition

The microbial community analysis results between the experimental groups are shown in Figure S3. The most abundant phyla were Firmicutes, Bacteroidetes, Actinobacteria, and Verrucomicrobia. At the order level, 10 taxa including Lactobacillales and Bifidobacteriales were found in all samples. Interestingly, *Akkermansia* (genus level) was detected relatively low in the DSS group.

**Figure 3:**
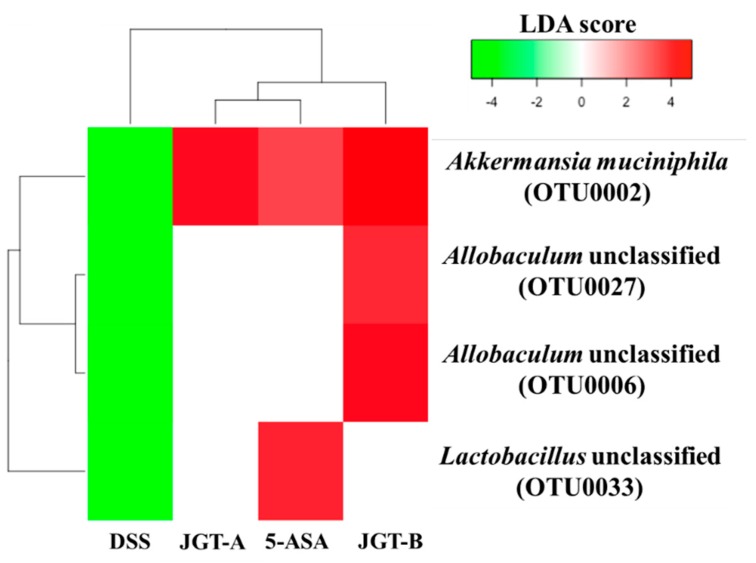
Heatmap analysis of OTUs that were significantly decreased by DSS treatment but significantly increased by treatments based on LEfSe results (LDA > 3, *p* < 0.05). DSS and control groups were compared to identify OTUs that were significantly decreased by DSS. DSS and treatment groups were compared to identify OTUs that were significantly increased by treatments.

As a result of the comparison between DSS and the control groups, there were 19 and 25 OTUs that significantly decreased and increased by DSS treatments, respectively (LDA score > 3, *p* < 0.05). Among them, OTUs that decreased by DSS but increased by 5-ASA or JGT include *Akkermansia*, *Allobaculum*, and *Lactobacillus* (Figure 3). These results suggest that 5-ASA or JGT treatment can restore microbial changes in the gut caused by DSS induction. The increased abundance of *Lactobacillus* was only observed in the 5-ASA group compared to that in the DSS group. The increase of *Akkermansia* by 5-ASA and JGT may support that these treatments recovered epithelial tissue damages caused by DSS (Figure 1A). *Akkermansia* is known as a mucin-degrading bacterium that uses mucin as a source of energy [44]. However, the beneficial effect of *Akkermansia* on colitis is associated with an increase of goblet cells [45]. The reduction of *Akkermansia* abundance following antibiotic treatment is accompanied by reduced mucolysis and a reduction in the expression of gene Muc2 encoding major mucin of the colonic mucus in colonic tissues [46]. According to Kang et al. [47], extracellular vesicles from *Akkermansia muciniphila* can prevent DSS-induced colitis. In addition, previous studies have shown that *Akkermansia* is closely related to host immunity and is associated with the proliferation of anti-inflammatory regulatory T cells [48,49,50]. 

**Figure 4:**
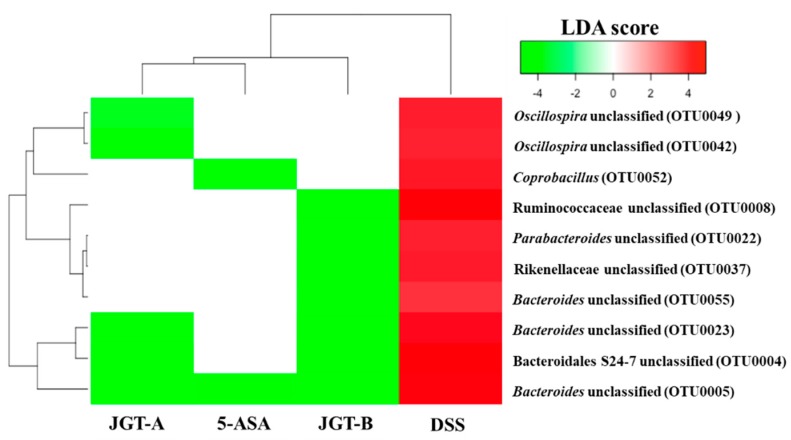
Heatmap analysis of OTUs that were significantly increased by the DSS treatment but significantly decreased by the treatments based on LEfSe results (LDA > 3, *p* < 0.05). The DSS and control groups were compared to identify OTUs that were significantly increased by DSS. DSS and treatment groups were compared to identify OTUs that were significantly decreased by treatments.

On the other hand, OTUs that were increased by DSS but decreased by the 5-ASA or JGT treatment include Bacteroidales S24-7, Ruminococcaceae, and Rikenellaceae, and the genera *Bacteroides*, *Parabacteroides*, *Oscillospira*, and *Coprobacillus* (Figure 4). The administration of 5-ASA or JGT significantly decreased Bacteroides known to have beneficial protective effect against IBD [51,52,53]. However, many previous studies reported that the relative high abundance of *Bacteroides* is associated with colitis onset [54,55]. Four S24-7 OTUs were not initially affected by DSS, but showed a significant increase after the 5-ASA and JGT treatment, suggesting that S24-7 may have played an important key role in improving damages caused by DSS. The bacteria belonging to S24-7 are unculturable, but it has been reported that S24-7 have abilities to use carbohydrate and can be divided into three groups based on the types of the carbohydrates (α-glucans, complex plant cell wall glycans, or host-derived glycans) [56]. Thus, some species of S24-7 may have provided beneficial substances through metabolizing 5-ASA or JGT. Some species of the genus *Oscillospira* increased due to DSS treatment in this study. While this genus is under-studied due to the difficulties in culturing, the previous metagenomic study suggested this bacteria could produce butyrate and was negatively associated with intestinal inflammation [57]. As culturing methods are being developed and the full length 16S rRNA gene database is expanding, future studies may provide a better understanding of this bacteria at the species-level.

Apart from these, there were 22 OTUs that were not affected by the DSS treatment but showed differential abundance by the 5-ASA or JGT treatment including the above-mentioned families and genera (Table S1), suggesting that the effects of 5-ASA and JGT are likely to be species specific.

### 3.4. JGT Treatment Changes Metabolites of Serum

**Figure 5:**
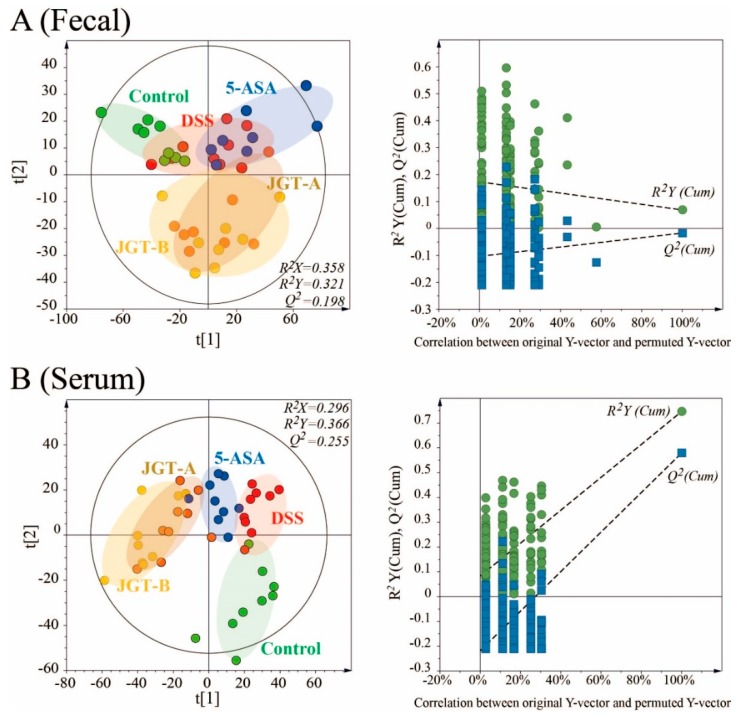
PLS-DA scores plots for control, DSS-induced colitis, 5-ASA, and JGT treated groups derived from GC/MS data of fecal (**A**) and serum (**B**) samples of rats. These PLS-DA models were validated by a permutation test (*n* = 200) and CV-ANOVA for assessing reliability.

To investigate the metabolic profiles in fecal and serum among the experimental groups, supervised partial least squares-discriminant analysis (PLS-DA) score plots were applied (Figure 5). The groups were not fully distinguishable in the PLS-DA score plot from the fecal samples (*R*^2^*X* = 0.358, *R*^2^*Y* = 0.321, *Q*^2^ = 0.198 *p*[CV-ANOVA] = 0.00301), indicating that the metabolite profiles among the groups were not significantly different. However, a clear separation among the groups was observed in the score plot from the serum samples (*R*^2^*X* = 0.296, *R*^2^*Y* = 0.366, *Q*^2^ = 0.255, *p*[CV-ANOVA] = 0.00011), suggesting that metabolites of the serum could be altered by colitis induction or JGT treatment. The permutation test supported the validity of this PLS-DA model. To find metabolites responsible for the classification of this PLS-DA model, the parameter of variable importance in projection (VIP) was determined. A potential metabolic biomarker was selected based on the value of VIP > 1.0 and the critical *p* value from the Student’s *t*-test. The DSS group was characterized by higher serum levels of lactic acid, alanine, glycine, 2-aminobutanoic acid, valine, leucine, isoleucine, proline, threonine, phenylalanine, tryptophan, myristic acid, 1-monopalmitine, 2-palmitoylglycerol, and glycerol monostearate but lower levels of glutamine and arachidonic acid in serum compared to the control group (*p* < 0.05) (Table 1). Interestingly, the JGT groups exhibited opposite metabolite patterns, except for valine and isoleucine compared to the DSS group. These results suggest that JGT treatment can restore metabolic changes caused by DSS induction.

The metabolites provide important information regarding small molecules that are either produced or modified by the gut microbiota that affect mucosal responses [58]. Additionally, changes in the levels of metabolites may reveal important clues about the severity of colitis [59]. In the present study, although gut metabolites were altered, there were no significant differences in gut metabolites between the groups. However, metabolites including most amino acids that were altered by colitis induction became similar to those of the control group after JGT administration. Numerous studies have shown that metabolic changes occur in IBD patients, altering the serum profile of amino acids [60,61,62,63]. However, the pattern of changes in individual amino acids varies from study to study.

In the present study, the levels of glutamine significantly decreased in the serum of the DSS group compared to those in the control group. The levels of glutamine significantly increased after the administration of 5-ASA and JGT compared to those in the DSS-induced colitis group. Some studies have reported that glutamine is associated with pro-inflammatory cytokines in colitis. According to a previous study [64], the post-treatment of glutamine can attenuate an inflammatory response in DSS-induced acute colitis by suppressing Th17-associated cytokine expressions and decreasing pro-inflammatory cytokine production in the gut. Hou et al. [65] reported that glutamine can significantly suppress the NF-κB expression in the gut of colitis mice compared to the control without glutamine treatment. According to Coëffier et al. [66], glutamine can increase the production of anti-inflammatory cytokine (IL-10) and decrease the production of pro-inflammatory cytokines (IL-6 and IL-8) in human intestinal mucosa.

Recent studies have indicated that the immunosuppressive function and antioxidant properties of tryptophan can help treat IBD. Tryptophan is involved in the regulation of the immune system through the interactions between IFN-γ and tryptophan-degrading enzyme [67]. Kim et al. [68] reported that tryptophan supplementation can reduce inflammation and enhance the rate of recovery in DSS-induced colitis. Alexeev et al. [69] reported that tryptophan levels significantly increased in the colons of colitic mice. In the present study, tryptophan was not detected in the fecal samples, but the levels of tryptophan in the serum decreased after the JGT treatment. Tryptophan can be directly transformed by the gut microorganisms into indole and its derivatives, which have been suggested as promising substances for colitis treatment [70]. However, the precise microbial enzyme pathways and their presence and activity in the gut microbiota have yet to be elucidated.

Several studies have reported that the incidence of colitis is correlated with the increase of lactic acid concentration in feces [71] and serum [72]. In the present study, lactic acid concentrations significantly increased in the serum samples after colitis induction. However, they showed no significant difference in the fecal samples. After colitis induction, Lactobacillus known to produce lactic acid, significantly decreased. In addition, *Allobaculum* significantly decreased by the DSS treatment but increased with the JGT treatment. *Allobaculum* has been reported as potential probiotic bacteria that utilize prebiotics and bring benefits to the host [73] and are also known as active lactate utilizers/butyrate producers in murine [74]. These results suggest that the increase in the concentration of lactic acid in serum might be due to the destruction of the colon mucosal cell lining or the alteration of the host metabolic pathway rather than due to the increase of lactic acid producing bacteria or a decrease of lactic acid utilizing bacteria in the gut.

### 3.5. Correlation Analysis

**Figure 6:**
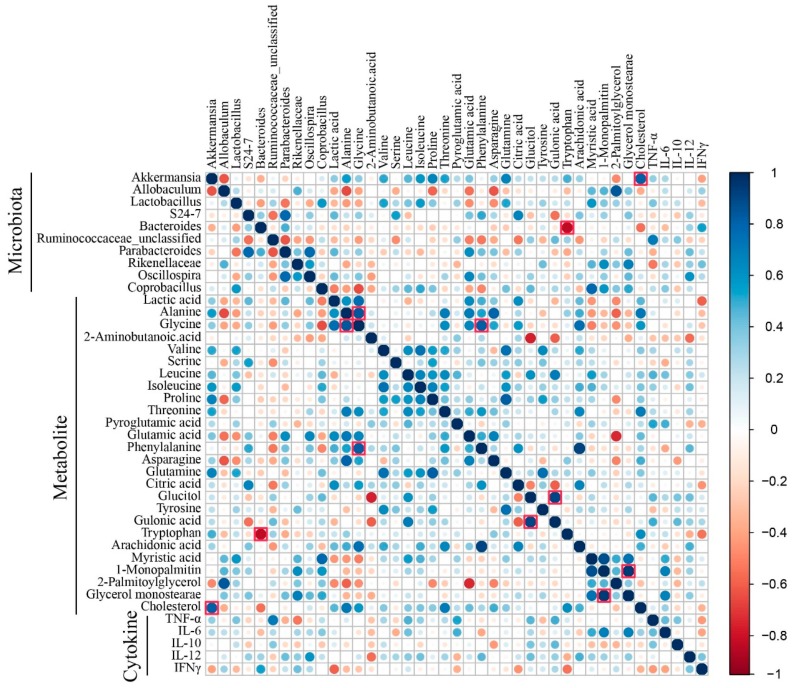
Heat map derived from the correlations among the altered gut microbiota, disturbed metabolites in serum, and changed cytokine levels. The correlation matrixes were formed based on Spearman’s rank correlation coefficients. The correlation coefficients greater than 0.8 in the heat map were indicated by red square borders.

The correlation matrixes were formed based on Spearman’s rank correlation coefficients to explore functional relationships among the altered gut microbiota, disturbed metabolites in serum, and changed cytokine levels (Figure 6). Glycine was positively correlated with alanine (rho = 0.84, *p* < 0.001, *q* = 0.02) and phenylalanine (rho = 0.82, *p* < 0.001, *q* = 0.025). Monopalmitin was positively correlated with glycerol monostearate (rho = 0.94, *p* < 0.001, *q* = 0.005) and myristic acid (rho = 0.87, *p* < 0.001, *q* = 0.015). Gulonic acid was positively correlated with glucitol (rho = 0.90, *p* < 0.001, *q* = 0.01). *Akkermansia* displayed strong correlations with increased levels of cholesterol (rho = 0.81, *p* < 0.001, *q* = 0.03), and *Bacteroides* displayed strong correlations with decreased levels of tryptophan (rho = −0.83, *p* < 0.05, *q* = 0.035). The correlation between the altered gut microbiota and fecal metabolites was shown in Figure S4. However, specific metabolite changes by each microbial taxon in the gut microbiota need further evaluation.

Although many treatment strategies for colitis have been clinically examined, new therapeutic approaches are needed because current therapies show no effect in many patients or have severe side effects. Five-ASA, also known as mesalazine or mesalamine, is a highly effective treatment for both ulcerative colitis and for Crohn’s disease [6]. However, the side effects, including headache, nausea, abdominal pain, and fever, are common. Five -ASA treatment often require multiple doses and many pills every day because the general response rate is only 70–80% and the relapse rate is largely different [75]. Therefore, it is necessary to search for strategies to treat IBD safely and effectively. In the present study, the therapeutic effect of JGT was similar to that of 5-ASA, suggesting that JGT can be a promising herbal medicine candidate for colitis treatment

Previous studies have reported that some polyphenols, such as gallic acid and glycyrrhizin, in the formula have effects on IBD [76,77,78,79]. However, oriental medicine emphasizes the achievement of health by maintaining an equilibrium between various systems and functions of an individual due to the complex mixture rather than a single compound. From this point of view, JGT can be effective for treating colitis by restoring dysbiosis of the gut bacterial community. Many studies have shown structural changes of gut microbiota induced by herbal medicine. Some medicinal herbs can increase the proportion of *Akkermansia* with a concomitant beneficial effect on the host metabolism [80,81,82]. *Gegen Qinlian Decoction* treatment can enrich amounts of beneficial bacteria, such as *Faecalibacterium* spp. [83]. Likewise, a complex mixture including fibers, polyphenols, and polysaccharides present in JGT can affect gut bacteria. *P. lactiflora* root steam distillate has been shown to possess high growth-inhibiting activity against nine harmful gut bacteria including *Clostridium difficile* and *Escherichia coli* [84]. According to Peng et al. [85], the administration of *P. lactiflora* extract could not only correct the changed taxonomic composition, but also increase the relative abundances of beneficial bacteria in the gut of arthritis rats. The compounds ingested as herbal extracts can reach the gut due to their low digestibility and influence the human microbial community by stimulating or inhibiting the growth of certain microorganisms [86]. The altered microbiota in the gut by herbal extracts can affect human health through microbial metabolites [83]. The results of the current study suggest that JGT can exert anti-inflammatory effects by regulating microbiota in DSS-induced colitis. However, in this study, it is unclear whether the effects on the microbiota are directly caused by JGT, or indirectly through the effects of JGT on the inflammatory state of the gut. To determine that the effects on gut microbiota are directly caused by JGT, further studies are needed to observe the changes in the gut of the control group administered JGT that did not induce colitis.

## 4. Conclusions

In this study, JGT treatment significantly remitted DSS-induced colitis by improving DAI scores, reducing inflammatory cytokines, and restoring changed microbiome and metabolome, suggesting that JGT is a promising herbal medicine candidate for colitis treatment. However, some issues such as how JGT changes gut microbiome and metabolites and how JGT regulates immune function need to be clarified. Further investigations are needed to explain the specific mechanisms that support the present observations.

## Figures and Tables

**Table 1:** Metabolic effects in serum of DSS-induced colitis and 5-ASA or JGT administration.

Metabolites	RT	RI	DSS/Control		5-ASA/DSS		JGT (150 mg/kg)/DSS	
			↑/↓ ^1^	*p* ^2^	↑/↓	*p*	↑/↓	*p*
Lactic acid	6.49	915	↑	*	↓	**	↓	***
Alanine	7.11	1038	↑	***	↓	*	↓	***
Glycine	7.35	1002	↑	**	↓	*	↓	***
2-Aminobutanoic acid	8.15	1137	↑	*	↓	ns	↓	**
Valine	8.80	1172	↑	*	↑	***	↑	***
Serine	9.39	1158	↓	ns	↑	**	↑	**
Leucine	9.60	1272	↑	***	↑	ns	↓	**
Isoleucine	9.92	1272	↑	*	↑	*	↑	***
Proline	9.97	1258	↑	***	↓	ns	↓	***
Threonine	11.21	1357	↑	***	↑	ns	↓	**
Pyroglutamic acid	12.96	1466	↑	ns	↑	ns	↑	ns
Glutamic acid	14.07	1612	↑	ns	↓	**	↓	***
Phenylalanine	14.20	1711	↑	***	↓	ns	↓	***
Asparagine	14.69	1745	↓	ns	↑	**	↑	***
Glutamine	15.81	1845	↓	**	↑	**	↑	***
Citric acid	16.36	1944	↑	ns	↓	*	↓	*
Glucitol	17.22	2066	↓	ns	↑	ns	↑	*
Tyrosine	17.52	2008	↓	***	↑	*	↑	ns
Gulonic acid	18.04	1981	↓	ns	↑	ns	↑	**
Tryptophan	20.11	2257	↑	***	↓	ns	↓	**
Arachidonic acid	21.30	2417	↓	**	↓	ns	↑	ns
Myristic acid	21.52	2382	↑	*	↑	ns	↑	ns
1-Monopalmitin	23.02	2581	↑	*	↑	ns	↑	ns
2-Palmitoylglycerol	24.17	2581	↑	*	↑	ns	↑	ns
Glycerol monostearae	24.42	2780	↑	*	↑	ns	↑	ns
Cholesterol	27.71	2654	↓	ns	↑	*	↑	*

^1^ Arrows (↑and↓) represent decrease or increase in metabolite levels in DSS group compared to control group and in 5-ASA or JGT treatment groups compared to DSS group; ^2^ The statistical outcome was summarized within the table (*t* test: *p* value). Symbols (*) indicate significant difference between groups. * *p* < 0.05; ** *p* < 0.01; *** *p* < 0.001; NS = not significant.

## Supplementary Materials

The following are available online at https://www.mdpi.com/2218-1989/9/10/226/s1, Figure S1: Workflow of animal experiment, Figure S2: Alpha diversity indices boxplot, including community richness (ACE, Chao 1, and OTUs) and diversity (Shannon, Simpson), are different among groups, Figure S3: Composition of microbiota at phylum level, order level, and genus level, Figure S4: Heat map derived from correlations among altered gut microbiota and fecal metabolites. Correlation matrixes were formed based on Spearman’s rank correlation coefficients, Table S1: Operational taxonomic units (OTUs) that were not affected by DSS treatment but showed significant differential abundance by 5-ASA and JGT treatments.
